# Mechanical Circulatory Support Reduces Directly Recorded Cardiac Sympathetic Nerve Activity in Ovine Acute Myocardial Infarction

**DOI:** 10.1016/j.jscai.2025.102642

**Published:** 2025-03-25

**Authors:** Tania Warnakulasuriya, Bindu George, Nigel Lever, Rohit Ramchandra

**Affiliations:** a Manaaki Manawa – The Centre for Heart Research and the Department of Physiology, University of Auckland, Auckland, New Zealand; b Department of Physiology, Faculty of Medicine, University of Kelaniya, Kelaniya, Sri Lanka; c Auckland District Health Board (Te Whatu Ora Health New Zealand Te Toka Tumai Auckland), Auckland, New Zealand

**Keywords:** arrhythmia, cardiac sympathetic nerve activity, mechanical circulatory support

Acute myocardial infarction (AMI) of the left ventricle activates cardiac sympathetic nerve activity (CSNA) as a compensatory mechanism to improve tissue perfusion.^1^ An elevation in CSNA increases heart rate and myocardial contractility, both of which operate to improve cardiac output. Although this may be beneficial in the short term, continued activation of CSNA is detrimental to cardiac recovery as increases in CSNA can result in fatal cardiac arrhythmias and sudden cardiac death. This elevation in CSNA can propel AMI to incipient cardiogenic shock, and once established, the cardiogenic shock downward cascade, if there is no intervention.

Mechanical circulatory support (MCS) is currently used in the management of left ventricular systolic dysfunction following AMI. Previous studies have indicated that MCS can reduce ventricular strain and improve coronary blood flow (CoBF).^2^ A preclinical study has suggested that MCS using Impella can also reduce induction of atrial arrhythmias in a porcine model of myocardial infarction^3^ although this has not been directly tested in a clinical cohort. While there is good evidence that heightened CSNA can elevate arrhythmia risk, how CSNA is modulated during MCS is not known. We tested the hypothesis that MCS using Impella would inhibit CSNA in an ovine model of AMI.

## Methods

All experiments were approved by the Animal Ethics Committee of the University of Auckland (AEC24524). This study was conducted on adult female Romney sheep (N = 13; range, 50-71 kg). The sheep were fasted for 24 hours before induction of anesthesia. Following anesthesia, surgical instrumentation was carried out to measure arterial pressure (solid-state catheter in femoral artery), CoBF (Transonic flow probes; 6PS), and CSNA (custom electrodes placed in the inferior cardiac nerve).^4^ CSNA was analyzed as the number of spikes crossing a threshold selected above the noise level in each animal as previously described.^4^ The spike numbers were averaged for the heart interval duration to calculate CSNA. Following a baseline period, the left coronary artery was embolized under fluoroscopy guidance.

### Induction of AMI

**Figure 1:**
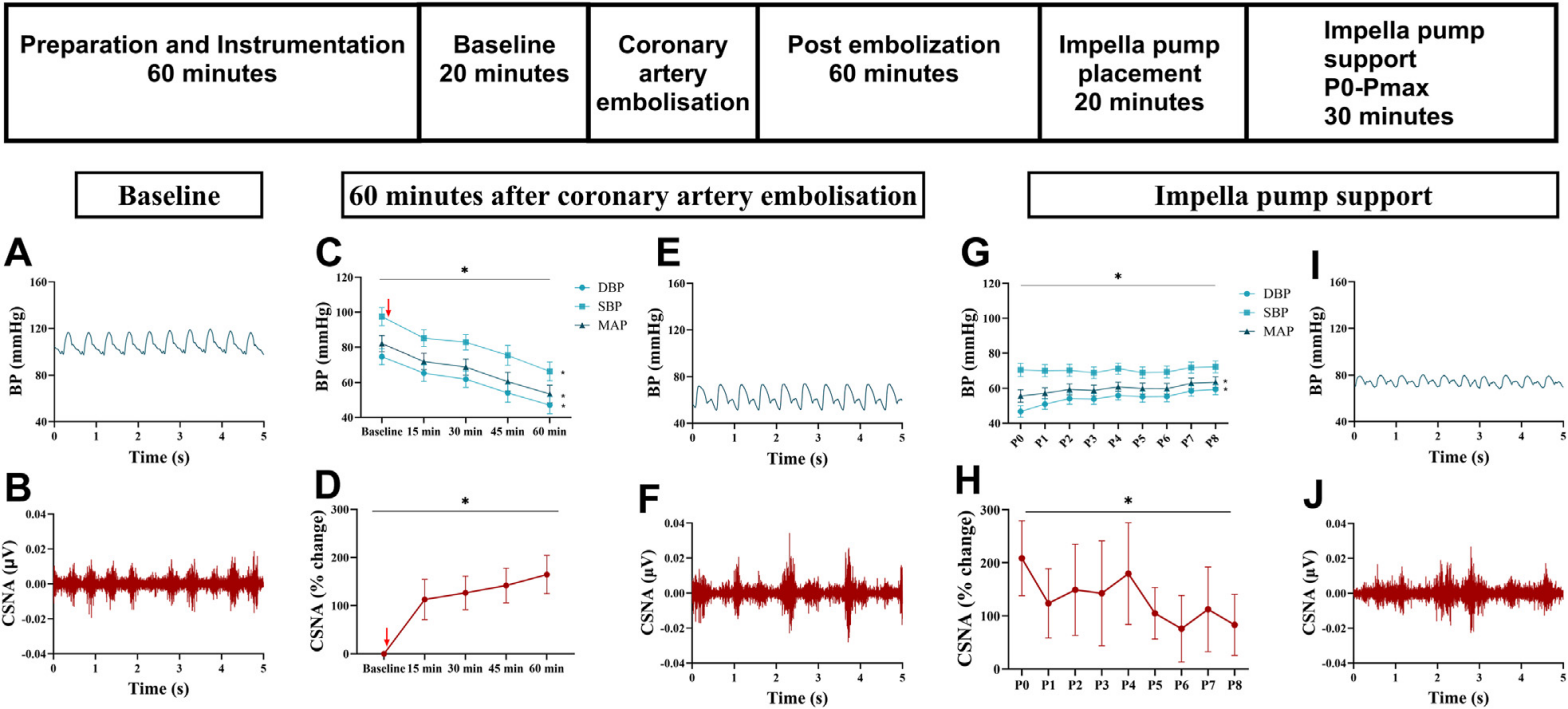
**Changes in CSNA with mechanical circulatory support.** The top panel shows the experimental protocol illustrating the procedures performed. Representative raw data traces of blood pressure (BP; **A**) and cardiac sympathetic nerve activity (CSNA; **B**) in 1 animal before embolization of the left main coronary artery. (**C**) The changes in diastolic blood pressure (DBP), mean arterial pressure (MAP), and systolic blood pressure (SBP; n = 13) and (**D**) percentage change in CSNA from baseline (n = 7) after embolization of the left coronary artery. ∗*P* < .05 (1-way ANOVA). The red arrow indicates embolization of the coronary artery. Representative raw data traces of BP (**E**) and CSNA (**F**) at 60 minutes after embolization of the left main coronary artery. (**G**) Changes to BP and (**H**) percentage change in CSNA (n = 7) during incremental pump support (P0-P8). Representative raw data traces of BP (**I**) and CSNA (**J**) during maximal pump support using the Impella device. ∗*P* < .05 (1-way ANOVA for pump levels). All significance tests are 2-tailed, and significance was accepted at *P* < .05.

The left main coronary artery was cannulated using an 8F AL2 angiography catheter inserted through the 6F introducer catheter placed in the right femoral artery (Cordis) under fluoroscopic guidance. Once the position was confirmed, 1.5-2.0 mL polystyrene latex microspheres (45 μm; Polysciences) were infused into the coronary artery. A change in the ST segment (elevation or depression) and T wave (inversion) were noted as an indication of a successful embolization. If the drop in blood pressure was not sustained, a smaller dose of microspheres was infused into the coronary arteries (within the first 10 minutes) until there was a sustained reduction in blood pressure. At 60 minutes after the embolization, the Impella pump was inserted (Figure 1).

### Insertion of Impella CP

The implantation of Impella CP (Abiomed Europe GmbH) was carried out per the manufacturer’s recommendations through access of one carotid artery. Using echocardiography, the Impella inlet position was adjusted to be in the midventricular cavity, with the outlet in the ascending aorta. The Impella controller recorded the pressure waveform, and the pressure wave was confirmed to be aortic in origin. The pump was started at level 1 (P1). Color Doppler was used to verify the inflow and outflow areas, and the position was adjusted as required. Once the positioning was satisfactory, the pump flow rate was increased every 2 minutes until level 8 or the highest level at which suction was noted. P0 was recorded when the pump was placed in the ventricle. Two minutes of continuous data were averaged at each stage of cardiac pump support. We note that owing to technical issues, we could not maintain CSNA recordings in 6 animals and CoBF waveforms in 7 animals. This included deterioration of the signal over time or distortion due to electrical noise from the Impella pump. We did not believe there was a bias in terms of which animals had successful recordings of either variable. Data are presented as the mean ± SEM. Statistical tests were conducted using SPSS version 28 (IBM Corp).

## Results

### Effect of left coronary artery embolization

Injection of microspheres into the coronary artery decreased mean arterial pressure by 28.6 ± 4.1 mm Hg at 60 minutes after embolization (n = 13) (Figure 1). The ejection fraction decreased from 73.7% ± 1.8% to 30.1% ± 2.3% (1-way ANOVA; *P* < .001). The decrease in arterial pressure was associated with a significant increase in CSNA such that at the 60-minute time point, CSNA increased by 164.6% ± 39.8% (1-way ANOVA; *P* < .05).

### Effect of left ventricular support

Impella MCS increased the mean arterial pressure from 54.3 ± 3.1 to 63.5 ± 3.1 mm Hg (n = 13) (Figure 1) at pump level P8 (1-way ANOVA P0-P8; *P* < .001). Incremental pump support resulted in a significant decrease in CSNA (n = 7) with 47.3% ± 6.9% reduction at P8 compared with that at P0 (1-way ANOVA; *P* < .001). Consistent with the reduction in CSNA, CoBF (n = 7) improved by 22.7 ± 6.8 mL/min at P8 compared with that at P0 (1-way ANOVA; *P* = .041).

## Discussion

To our knowledge, this study is the first to directly record CSNA during MCS, and we report a significant reduction in directly recorded CSNA levels with Impella circulatory support. Microembolization of the coronary arteries results in a substantial elevation of CSNA, consistent with a previous study that used a coronary obstruction model of AMI.^1^ This previous study in the conscious state did not observe a consistent drop in pressure,^1^ and our magnitude of CSNA increase was greater, suggesting that AMI with cardiogenic shock may further elevate CSNA. It is known that this elevation in cardiac sympathetic drive increases myocardial oxygen consumption and can elevate arrhythmogenic risk. Our data suggest this significant reduction in CSNA with MCS may offset the proarrhythmogenic state and may underlie the decreased incidence of arrhythmias observed with MCS in another study.^5^ It must be noted that mechanical irritation of the left ventricle can cause arrhythmias with Impella use, and hence, no studies have examined whether arrhythmia risk is altered with MCS. Increased CSNA can also cause intense coronary vasoconstriction, and our data indicate that a reduction in CSNA is associated with an improvement in directly recorded CoBF consistent with other reports of MCS.^2^ It is important to note that vasoactive medication as used clinically would further alter myocardial oxygen consumption and CSNA although this was not examined in this study. Another limitation of this study is that we did not measure plasma catecholamine levels to enable comparison in clinical subjects.

Taken together, our data indicate that the beneficial effects of Impella may be mediated in part by the inhibition of cardiac sympathetic drive as we have now shown with direct recordings.

## References

[1] Jardine D.L., Charles C.J., Ashton R.K. (2005). Increased cardiac sympathetic nerve activity following acute myocardial infarction in a sheep model. J Physiol.

[2] Sakata T., Mavropoulos S.A., Mazurek R. (2024). Reduction of left ventricular diastolic pressure as a key regulator of infarct coronary flow under mechanical left ventricular support. J Physiol.

[3] Ishikawa K., Watanabe S., Lee P. (2018). Acute left ventricular unloading reduces atrial stretch and inhibits atrial arrhythmias. J Am Coll Cardiol.

[4] Watson A.M., Hood S.G., Ramchandra R., McAllen R.M., May C.N. (2007). Increased cardiac sympathetic nerve activity in heart failure is not due to desensitization of the arterial baroreflex. Am J Physiol Heart Circ Physiol.

[5] Berg D.D., Singal S., Palazzolo M., CCCTN Investigators (2024). Modes of Death in Patients with Cardiogenic Shock in the Cardiac Intensive Care Unit: A Report from the Critical Care Cardiology Trials Network. J Card Fail.

